# Tailoring Cellulose Derivative Gel Matrices for *Bacillus subtilis* Delivery: Effects of Polymer Molecular Weight on Stability and Biocontrol

**DOI:** 10.3390/gels12050366

**Published:** 2026-04-27

**Authors:** Petya Tsekova, Nasko Nachev, Iliyana Valcheva, Donka Draganova, Mariya Spasova, Olya Stoilova

**Affiliations:** 1 Laboratory of Bioactive Polymers, Institute of Polymers, Bulgarian Academy of Sciences, 1113 Sofia, Bulgaria; cekovapetya@polymer.bas.bg (P.T.); nachev_n@polymer.bas.bg (N.N.); mspasova@polymer.bas.bg (M.S.); 2 National Centre of Excellence Mechatronics and Clean Technologies, 8 Blvd. Kliment Ohridski, 1000 Sofia, Bulgaria; 3 Biodinamika Ltd., 4000 Plovdiv, Bulgaria; valchevailiana1@gmail.com (I.V.); donkadraganova@gmail.com (D.D.)

**Keywords:** cellulose derivative films, *Bacillus subtilis*, biohybrid films, immobilization, swelling, antifungal activity, biocontrol

## Abstract

Cellulose-derived gel films are promising matrices for the immobilization and delivery of beneficial microorganisms in sustainable plant protection. This study evaluated the effects of polymer molecular weight and chemical structure on the physicochemical properties and biocontrol performance of hydroxyethyl cellulose (HEC) films of low, medium, and high molecular weight, as well as sodium carboxymethyl cellulose (CMC-Na), loaded with *Bacillus subtilis*. The films were characterized in terms of morphology, swelling behavior, mechanical properties, microbial viability, and antifungal activity against *Fusarium avenaceum* and *Alternaria solani*. Increasing HEC molecular weight produced progressively denser and more homogeneous gel networks, resulting in improved structural integrity, whereas CMC-Na formed dense but less stable networks. Swelling studies at pH 4, 7, and 9 showed high water uptake for all HEC systems, with enhanced structural stability observed in high-molecular-weight films, whereas CMC-Na dissolved rapidly under all conditions. Mechanical testing further confirmed that increasing molecular weight enhanced stiffness and tensile strength but reduced flexibility. Immobilized in gel matrices, *B. subtilis* remained viable after 12 months of storage and rapidly reactivated after rehydration. All biohybrid films inhibited fungal growth, with stronger formulation-dependent responses against *F. avenaceum* than against *A. solani*. In general, polymer molecular weight and structure were identified as key parameters controlling network organization, hydration behavior, mechanical performance, and biological functionality. These findings highlight the potential of cellulose-derived gel matrices as tunable carriers for microbial biocontrol applications.

## 1. Introduction

The imperative to develop sustainable agricultural practices has catalyzed the search for effective biological alternatives to synthetic pesticides and fertilizers. Fundamental to advancing this eco-friendly transition is the utilization of beneficial microorganisms, known as biological control agents (BCAs), which offer a dual advantage: suppressing phytopathogens and enhancing plant vigor through direct and indirect mechanisms [1]. Within this category, plant growth-promoting rhizobacteria (PGPR) are particularly notable due to their ability to support soil health and plant defense through multiple pathways, including nutrient solubilization, phytohormone production, and ecological niche competition [2,3]. Among these, *Bacillus subtilis*, a Gram-positive, spore-forming soil bacterium, represents a highly effective PGPR model. Its biocontrol potential is attributed to the production of diverse antimicrobial compounds such as lipopeptides and polyketides, competitive colonization of plant-associated niches, and activation of plant defense responses via induced systemic resistance [4,5,6]. Importantly, the formation of resilient endospores provides a significant technological advantage, enabling resistance to environmental stresses such as heat, desiccation, and UV radiation, thereby facilitating formulation and long-term storage [7].

Despite these advantages, the field performance of *B. subtilis*-based bioinoculants remains inconsistent. This variability is largely attributed to the complexity of soil environments, where introduced microorganisms must compete with established microbial communities while simultaneously adapting to abiotic stress factors such as temperature fluctuations, osmotic stress, and UV exposure [8,9]. These conditions can significantly reduce microbial viability and metabolic activity after application, ultimately limiting biocontrol efficacy. Therefore, the development of advanced delivery systems is essential to protect microbial cells during storage and application, while enabling their gradual activation and sustained functionality in the rhizosphere. Among the available strategies, entrapment within polymeric matrices has emerged as a highly promising approach. An ideal carrier should be biodegradable, non-toxic, and capable of forming a protective yet permeable microenvironment that supports microbial survival and controlled release [10].

In our previous work, chitosan-based cast films were demonstrated to be effective carriers for *B. subtilis*, where polymer molecular weight was identified as a key parameter controlling mechanical integrity, swelling behavior, and biological performance [11]. In that system, the intrinsic cationic nature and antimicrobial activity of chitosan contributed not only to structural properties but also to the overall antifungal effect. However, such bioactive polymer matrices inherently combine polymer-driven and microbe-driven effects, making it difficult to distinguish the specific contribution of the encapsulated microorganism.

To address this limitation and further advance the design of microbial delivery systems, the present study focuses on cellulose-based polymers as alternative immobilization matrices. Unlike chitosan, cellulose derivatives are generally non-ionic or anionic and do not exhibit intrinsic antimicrobial activity, thereby providing a more neutral platform to evaluate the role of polymer structure in regulating microbial viability and function [12,13,14]. Natural polysaccharides such as cellulose and its derivatives are particularly attractive due to their biocompatibility, biodegradability, and tunable physicochemical properties.

Among them, 2-hydroxyethyl cellulose (HEC), a water-soluble non-ionic cellulose ether, is especially suitable for the development of microbial carriers. HEC exhibits excellent film-forming ability, high water retention capacity, and the capability to form hydrated networks under mild conditions, all of which are beneficial for maintaining microbial viability [15,16]. Importantly, the functional properties of HEC-based matrices—including mechanical strength, swelling behavior, and diffusion characteristics—are strongly dependent on polymer molecular weight. Variations in molecular weight influence chain entanglement density, network mesh size, and viscoelastic behavior, which in turn govern mass transfer processes and the release of encapsulated cells [17]. In this study, three HEC grades with different molecular weights (HEC-L, HEC-M, and HEC-H) were employed, alongside sodium carboxymethyl cellulose (CMC-Na) as an anionic reference polymer, to systematically investigate these structure–function relationships.

From a technological perspective, the fabrication method also plays a crucial role in determining the applicability of such systems. While advanced techniques such as electrospinning enable the production of highly structured materials, simpler and scalable methods are more suitable for agricultural applications. Solution casting represents a straightforward, cost-effective, and widely used technique for producing uniform polymer films. Upon solvent evaporation, cohesive films are formed that can act as protective reservoirs for bioactive agents. In the context of microbial immobilization, such films provide a physical barrier against environmental stress while allowing controlled hydration and gradual release of cells and metabolites into the surrounding environment, thereby supporting prolonged biological activity [18].

Previous studies have explored various polymeric systems for microbial entrapment. Chitosan-based matrices have demonstrated enhanced viability and antifungal performance of *B. subtilis* [19], while electrospun poly(3-hydroxybutyrate) fibers coated with cellulose derivatives have supported bacterial survival and growth [20]. Alginate-based systems are also widely used due to their mild gelation conditions, although they often suffer from limited mechanical stability and uncontrolled release profiles [21,22]. Compared to these systems, cellulose-based cast films offer a favorable combination of simplicity, structural stability, and tunable diffusion properties [23].

Nevertheless, the specific influence of cellulose molecular architecture—particularly molecular weight—on the long-term viability, germination, and sustained biological activity of immobilized *B. subtilis* remains insufficiently understood. Most existing studies focus primarily on encapsulation efficiency or short-term viability, while the critical post-application stages, including spore germination, vegetative growth, and prolonged antifungal activity, are rarely addressed.

Therefore, the aim of the present study is to develop cellulose-based biohybrid cast films as carriers for *Bacillus subtilis* and to systematically evaluate the effect of polymer type and molecular weight on film properties and biological performance. Upon hydration, the cellulose films transition into soft surface-associated gels, which regulate water uptake, spore activation, and metabolite diffusion. Particular emphasis is placed on long-term microbial activity and antifungal efficacy against selected phytopathogenic fungi. By decoupling polymer bioactivity from microbial function, this work provides new insights into the design of structurally controlled delivery systems for sustainable plant protection applications.

## 2. Results and Discussion

Cellulose derivative films were selected as biopolymer matrices due to their biodegradability, biocompatibility, and tunable physicochemical properties, which make them suitable carriers for microbial immobilization and controlled delivery systems [15,24]. All films were prepared at a fixed polymer concentration of 1% (*w*/*v*) to ensure comparability between formulations. Hydroxyethyl cellulose (HEC) of different viscosities (HEC-L: 124 cP; HEC-M: 248 cP; HEC-H: 2370 cP) and sodium carboxymethyl cellulose (CMC-Na: 226 cP) were selected to systematically evaluate the effect of polymer molecular weight and chain entanglement on film structure and performance. HEC was chosen as a non-ionic cellulose derivative, while CMC-Na represents an anionic polyelectrolyte, allowing comparison of neutral versus charged polymer networks [14]. The solution-casting procedure was performed according to a previously described method [11], and all films were prepared under identical laboratory conditions on a leveled surface to ensure uniform spreading of the polymer solution during drying. Although Petri dishes were used as casting substrates, the resulting films were easily removed after complete drying without structural damage (Figure S1, Supplementary Materials). In addition, the good reproducibility of the casting process was confirmed by the consistent thickness values obtained for each formulation. All obtained films were transparent, confirming homogeneous polymer distribution and successful film formation. HEC-based films exhibited high flexibility and elasticity, whereas CMC-Na films were noticeably stiffer and brittle, which is consistent with literature reports [25,26]. These differences reflect variations in intermolecular interactions, viscosity, and network organization, which are expected to strongly influence swelling behavior, mechanical properties, and the release dynamics of immobilized *Bacillus subtilis*.

### 2.1. Morphological Characterization of Cellulose Derivative Films

The surface morphology of cellulose derivative films, both without and with immobilized *Bacillus subtilis*, was examined by scanning electron microscopy (SEM). The bacteria-free films (Figure S2, Supplementary Materials) exhibited continuous, smooth, and defect-free surfaces, confirming the effectiveness of the solution-casting method in producing homogeneous cellulose derivative films. Distinct differences in surface architecture were observed as a function of polymer type and molecular weight (Figure S2, Supplementary Materials). Films based on low-molecular-weight HEC (HEC-L, Figure S2a) exhibited a relatively loose and slightly heterogeneous morphology, with minor surface irregularities indicative of lower chain entanglement density. In contrast, medium-molecular-weight HEC (HEC-M, Figure S2b) formed more uniform and continuous structures, suggesting improved intermolecular interactions and network organization. Sodium carboxymethyl cellulose (CMC-Na, Figure S2d) displayed a morphology comparable to HEC-M, likely due to its similar molecular weight range. However, its slightly less cohesive surface can be attributed to its ionic character and higher hydrophilicity, which promote rapid hydration and reduced intermolecular cohesion. High-molecular-weight HEC (HEC-H, Figure S2c) exhibited the most homogeneous and densely packed morphology, characterized by a smooth and continuous surface. This behavior reflects enhanced chain entanglement, stronger hydrogen bonding, and increased packing density associated with higher molecular weight. These observations are in agreement with previous studies reporting that cellulose derivatives form structurally coherent films, where molecular weight and polymer chemistry govern network density and surface organization [14,15,16,17]. The bacteria-free films demonstrate that polymer molecular weight and chemical structure govern surface compactness and network organization, which are critical for subsequent interactions with immobilized bioactive agents.

**Figure 1:**
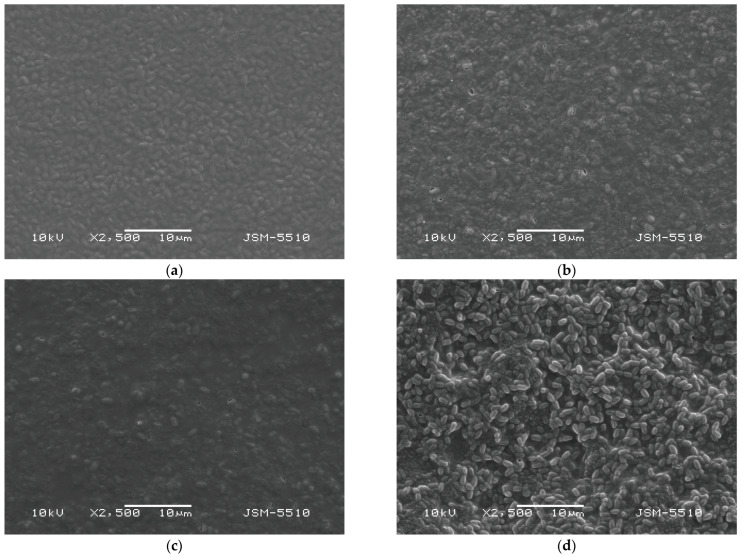
SEM micrographs of cellulose derivative films loaded with *Bacillus subtilis*: (**a**) HEC-L; (**b**) HEC-M; (**c**) HEC-H; (**d**) CMC-Na.

SEM micrographs of the biohybrid films (Figure 1) confirm the successful incorporation of *Bacillus subtilis* within cellulose derivative matrices while preserving overall structural integrity. The spatial distribution and visibility of the spores were strongly influenced by polymer type and molecular weight. In HEC-L films (Figure 1a), the surface exhibited a relatively heterogeneous and moderately porous morphology, with dispersed oval-to-rod-shaped features corresponding to partially exposed spores. This indicates a lower degree of matrix compactness and weaker physical confinement. With increasing molecular weight, the polymer networks became progressively more organized and denser. HEC-M films (Figure 1b) displayed a smoother and more continuous surface, with reduced roughness and less distinct spore features, suggesting improved embedding within the matrix. The HEC-H films (Figure 1c) exhibited the most homogeneous and compact morphology, characterized by a dense and continuous structure with minimal visible surface heterogeneity. In this case, spores were scarcely distinguishable, indicating effective immobilization and strong physical confinement within the highly entangled polymer network. In contrast, CMC-Na films (Figure 1d) showed numerous clearly visible rod-shaped structures attributable to *B. subtilis*. Their pronounced visibility is likely due to the relatively lower cohesion of the matrix, combined with its ionic nature and higher hydrophilicity, which reduce packing density and facilitate partial surface exposure. This behavior is comparable to that observed for HEC-M, supporting the notion that both molecular weight and chemical functionality contribute to matrix organization.

SEM analysis demonstrates a clear correlation between polymer architecture and immobilization behavior. As molecular weight increases, the transition from loosely organized to highly compact networks lead to progressively deeper embedding of the biological component. This structure-dependent immobilization has been reported in other polysaccharide-based delivery systems, where increased network compactness enhances retention of bioactive agents and limits their surface exposure [17]. Functionally, these structural differences have important implications: fewer compact matrices (e.g., HEC-L) may facilitate faster microbial release and interaction with the environment, whereas denser systems (e.g., HEC-H) provide enhanced protection and sustained retention, potentially improving long-term stability and controlled activity. The absence of cracks or phase separation in all formulations further confirms the good structural integrity of the prepared films. These findings are consistent with trends observed in related polysaccharide systems, including chitosan-based matrices, highlighting polymer molecular weight as a key parameter for tuning biohybrid film performance [11].

### 2.2. Swelling Behavior of Cellulose Derivative Films

**Figure 2:**
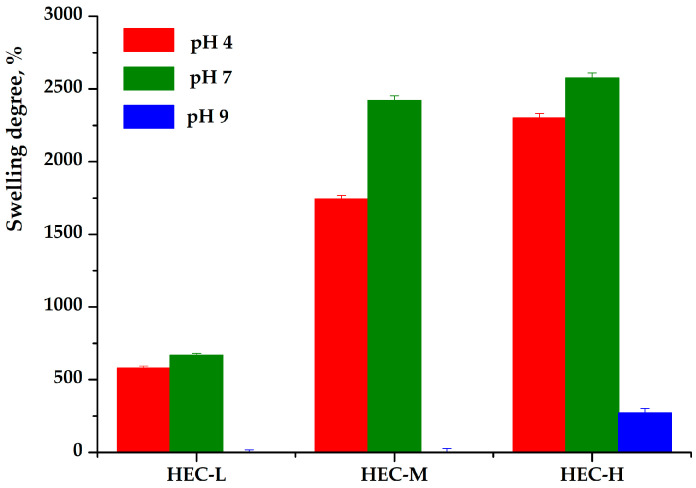
Swelling behavior of cellulose derivative films at different pH values.

The swelling behavior of the cellulose derivative films was investigated in buffer solutions at pH 4, 7, and 9 (Figure 2) to evaluate the combined effects of polymer type, molecular weight, and environmental conditions on hydration dynamics and structural stability.

Marked differences were observed among the tested systems, highlighting the critical role of polymer architecture. The non-ionic character of 2-hydroxyethyl cellulose (HEC) enables relatively stable behavior across a broad pH range, as its hydration is primarily governed by hydrogen bonding and water diffusion rather than ionization. In contrast, sodium carboxymethyl cellulose (CMC-Na), an anionic polyelectrolyte, exhibits pH-dependent behavior due to ionizable carboxymethyl groups, which influence chain conformation, electrostatic interactions, and solubility [27,28].

Under acidic conditions (pH 4), all HEC-based films demonstrated rapid water uptake followed by structural destabilization (Figure 2). HEC-L reached a swelling degree of approximately 580% within 30 min before disintegrating into gel-like fragments that gradually dissolved. HEC-M exhibited significantly faster and higher swelling (~1743% within 5 min), but rapidly lost structural integrity, indicating a weakly interconnected network. In contrast, HEC-H showed the highest swelling capacity (~2300% within 60 min) and, despite partial disintegration, maintained gel-like residues for several days, reflecting enhanced network cohesion. CMC-Na films dissolved almost instantaneously (<5 min), indicating high water sensitivity and lack of structural stability. At neutral pH (pH 7), swelling capacity increased further (Figure 2). HEC-L reached ~666% within 10 min, followed by rapid disintegration, while HEC-M and HEC-H exhibited significantly higher swelling (~2420% and ~2576%, respectively). Notably, only HEC-H retained gel integrity over extended periods, whereas CMC-Na again dissolved immediately. Under alkaline conditions (pH 9), all systems exhibited reduced stability (Figure 2). HEC-L and HEC-M disintegrated rapidly without forming stable swollen structures. HEC-H showed lower swelling (~270% at 20 min), yet preserved gel-like remnants for several days, demonstrating that high molecular weight contributes to structural persistence even under less favorable conditions. As in other media, CMC-Na dissolved rapidly regardless of pH.

The experimental findings clearly demonstrate that swelling behavior is governed by both molecular weight and polymer chemistry. For HEC systems, increasing molecular weight (HEC-L < HEC-M < HEC-H) enhances chain entanglement and intermolecular interactions, resulting in more cohesive networks capable of retaining large amounts of water while delaying dissolution. Conversely, lower-molecular-weight formulations form comparatively less stable networks that, although highly hydrophilic, lack the mechanical integrity required to maintain a swollen state. The behavior of CMC-Na differs fundamentally due to its ionic nature. Above the pKa of its carboxyl groups (~4.5–5), ionization leads to increased negative charge density along the polymer chains, promoting electrostatic repulsion and rapid network expansion. However, this also accelerates chain separation and dissolution, particularly in dilute systems, explaining the immediate disintegration observed across all pH conditions. Similar effects have been reported for cellulose-based hydrogels, where ionization-driven swelling is accompanied by reduced structural stability due to charge screening and osmotic effects.

A clear correlation can be established between swelling behavior and the morphological characteristics observed by SEM. The dense and homogeneous structure of HEC-H films supports the formation of a robust network that resists rapid dissolution and enables sustained gel formation. In contrast, the more open and less organized morphologies of HEC-L and HEC-M facilitate rapid water penetration and structural breakdown. The high solubility of CMC-Na is likewise consistent with its comparatively weaker intermolecular cohesion.

These findings are in agreement with recent literature on cellulose-derived materials, which emphasizes that swelling is controlled by the interplay between hydrophilicity, chain entanglement, and network density [14,15,16,17]. Non-ionic cellulose ethers such as HEC are known to form hydrogen-bonded networks with high water uptake capacity, while increased molecular weight improves structural stability and prolongs hydration lifetime. In contrast, CMC-based systems exhibit rapid hydration and dissolution due to their polyelectrolyte nature and strong affinity for water. Reported swelling values for similar systems range from several hundred to over 2000%, consistent with the results obtained in the present study [29].

In general, the results highlight that polymer molecular weight and chemical functionality serve as key design parameters for tuning hydration behavior. This structure–property relationship is particularly important for biohybrid systems, where controlled swelling and structural persistence directly influence microbial protection, release kinetics, and long-term functionality.

### 2.3. Mechanical Characterization of Cellulose Derivative Films

The mechanical behavior of the cellulose derivative films is strongly governed by polymer molecular weight and chemical structure [30], in agreement with the morphological and swelling characteristics discussed previously. The results demonstrate a clear transition from flexible, low-strength systems to rigid, highly stiff networks as molecular weight and ionic character increase.

**Figure 3:**
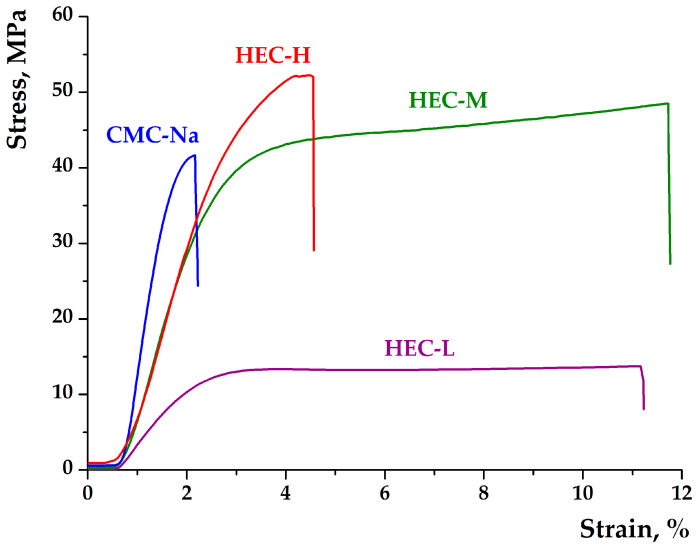
Stress–strain curves of cellulose derivative films.

HEC-L films exhibited the lowest Young’s modulus (867 MPa) and tensile strength (13.8 MPa), combined with relatively high elongation at break (11.2%). This indicates a loosely packed polymer network with limited chain entanglement, resulting in a highly deformable but mechanically weak structure. Such behavior is consistent with the more open morphology observed by SEM and the rapid swelling and disintegration reported for this formulation, confirming its low structural resistance. In contrast, increasing molecular weight significantly enhanced mechanical performance (Figure 3). HEC-M showed a marked increase in stiffness (2445 MPa) and tensile strength (48.6 MPa) while maintaining comparable elongation (11.7%), indicating a balanced structure with improved chain entanglement and intermolecular interactions without substantial loss of flexibility. This combination suggests an optimized network architecture that supports both mechanical stability and deformation capacity. HEC-H films demonstrated the highest tensile strength (52.2 MPa) and high stiffness (2370 MPa), but with a pronounced reduction in elongation at break (4.5%). This reflects a densely entangled and highly compact polymer network, where strong intermolecular interactions enhance mechanical resistance but limit chain mobility. This rigid structure is consistent with SEM observations showing homogeneous and compact morphology, as well as swelling results indicating delayed hydration and improved structural integrity. CMC-Na films exhibited the highest Young’s modulus (4666 MPa), confirming their highly rigid and stiff nature. However, this was accompanied by moderate tensile strength (41.2 MPa) and very low elongation (2.2%), indicating a brittle mechanical profile. This behavior can be attributed to the ionic character of CMC, where strong intermolecular associations and electrostatic interactions lead to a tightly packed network with limited ability to dissipate stress through chain deformation. As a result, the material resists deformation but fails at low strain.

Comparatively, the mechanical properties reveal two distinct behavioral regimes: HEC-based films, particularly HEC-L and HEC-M, provide more ductile and adaptable matrices, while HEC-H and CMC-Na form mechanically robust but significantly more brittle systems (Figure 3). These differences are directly reflected in their functional behavior, where flexible matrices facilitate rapid swelling and diffusion, whereas rigid structures promote controlled release and improved structural stability. Importantly, the higher integrity and stiffness of HEC-H and CMC-Na systems support sustained structural stability during hydration, while more flexible HEC-L matrices enable faster interaction with the surrounding environment but with reduced long-term stability.

These results confirm that molecular weight and polymer chemistry are key determinants of the mechanical profile of cellulose derivative films, enabling fine-tuning of material properties for targeted bioactive delivery systems.

### 2.4. Microbiological Purity, Viability, and Long-Term Stability of Biohybrid Films

Microbiological purity is essential to ensure the safety, stability, and reproducibility of biomaterial systems, as unintended microbial contamination may alter material properties, promote degradation, and compromise the performance of controlled-delivery applications [31]. In microbial delivery platforms, preservation of cell viability during processing and storage is equally critical, since desiccation, osmotic stress, and polymer–cell interactions may reduce survival and biological functionality. Previous studies on hydrogel and polysaccharide matrices have shown that polymer composition strongly influences microbial persistence and reactivation after rehydration [11]. However, long-term stability data for *Bacillus subtilis* immobilized in cellulose-derived films remain limited. Therefore, the present study evaluated microbiological purity, viability, and biological performance after 12 months of storage under ambient conditions.

**Figure 4:**
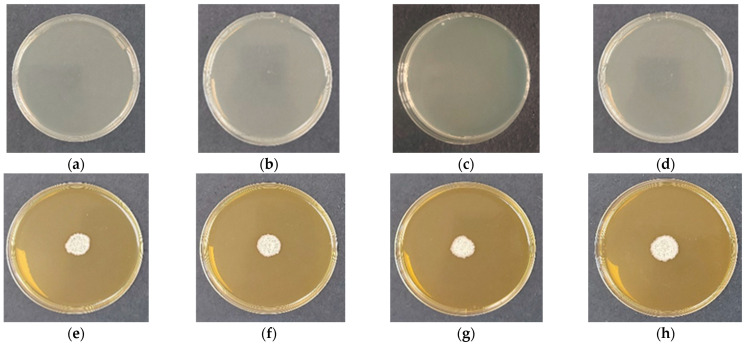
Microbiological purity and viability of cellulose derivative films after 48 h of incubation. (**a**–**d**) Bacteria-free control films: (**a**) HEC-L; (**b**) HEC-M; (**c**) HEC-H; (**d**) CMC-Na. (**e**–**h**) Films containing *Bacillus subtilis*: (**e**) HEC-L/*B. subtilis*; (**f**) HEC-M/*B. subtilis*; (**g**) HEC-H/*B. subtilis*; (**h**) CMC-Na/*B. subtilis*.

The microbiological purity of the cellulose derivative films was initially verified using control samples without incorporated bacterial spores. After 48 h of incubation on solid nutrient medium, no visible microbial growth was detected for any of the tested formulations (Figure 4a–d), confirming the sterility of the film-forming process and the absence of external contamination. This result demonstrates that the applied solution-casting methodology provides a controlled environment suitable for the preparation of biohybrid systems.

Following confirmation of sterility, the viability of immobilized *Bacillus subtilis* was subsequently evaluated. Upon contact with the moist nutrient surface, all films rapidly rehydrated and established a localized aqueous microenvironment favorable for spore germination and subsequent bacterial growth. After 48 h of incubation, clearly visible colonies were observed in all biohybrid samples (Figure 4e–h), indicating that the entrapment and film-forming procedures preserved spore viability. The results indicate that while all cellulose derivative matrices effectively preserve bacterial viability, the spatial organization and growth dynamics of *B. subtilis* are strongly influenced by polymer molecular weight and chemical structure. The correlation between SEM morphology, swelling behavior, and microbiological performance highlights the role of polymer architecture in modulating the microenvironment of immobilized cells. Importantly, none of the tested systems exhibited inhibitory effects on germination or vegetative growth, confirming that these materials act as biologically compatible carriers suitable for microbial delivery applications.

In order to assess long-term stability, the films were stored under ambient laboratory conditions (23 ± 2 °C; 45 ± 5% relative humidity), protected from direct sunlight, for a period of 12 months without controlled atmosphere. During this time, no visible changes in physical appearance or handling properties were detected. The films remained intact, flexible, non-adhesive, and free of secondary contamination.

**Figure 5:**
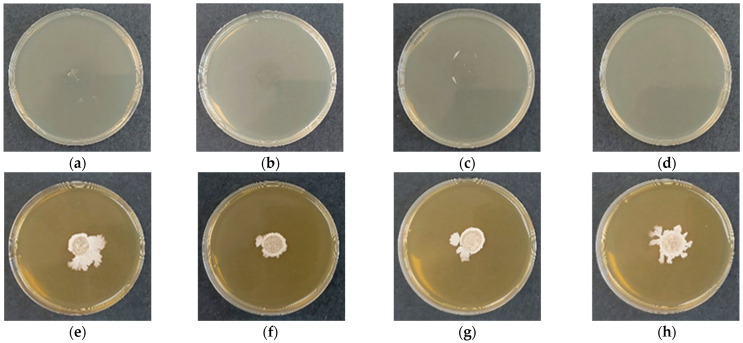
Microbiological purity and viability of cellulose derivative films after 12 months of storage under ambient conditions. (**a**–**d**) Bacteria-free control films: (**a**) HEC-L; (**b**) HEC-M; (**c**) HEC-H; (**d**) CMC-Na. (**e**–**h**) Films containing *Bacillus subtilis*: (**e**) HEC-L/*B. subtilis*; (**f**) HEC-M/*B. subtilis*; (**g**) HEC-H/*B. subtilis*; (**h**) CMC-Na/*B. subtilis*.

Microbiological purity after storage was confirmed by incubating bacteria-free control films on solid medium (Figure 5a–d), where no microbial growth was observed, indicating preservation of sterility over time. The viability of entrapped *B. subtilis* was evaluated under the same conditions (Figure 5e–h). Upon placement on TSA, the films rapidly absorbed moisture and underwent complete rehydration, followed by dissolution and infiltration into the agar matrix. No visible residues of the polymer remained at the contact interface, suggesting efficient matrix disintegration and release of the immobilized spores. After 48 h of incubation, well-defined bacterial colonies were observed for all formulations, confirming that spore viability was retained after prolonged storage. The colonies exhibited morphology characteristic of the strain, and signs of active sporulation were detected, indicating that the cells maintained normal physiological activity. This observation suggests that neither the entrapment process nor long-term storage induced detectable stress or loss of functionality.

These results demonstrate that cellulose derivative films provide a protective and biologically compatible microenvironment that preserves both sterility and microbial viability over extended periods. The strong correlation between polymer structure, hydration behavior, and microbial performance further supports the role of molecular weight and polymer chemistry as key parameters in the design of stable biohybrid delivery systems.

### 2.5. Antifungal Activity of Biohybrid Films

The antifungal activity of the developed cellulose derivative films was evaluated against *Fusarium avenaceum* and *Alternaria solani*, two economically important phytopathogens responsible for significant crop losses in cereals production [32]. These fungi were selected due to their contrasting infection strategies and sensitivity to biological control agents, enabling a more comprehensive assessment of the biocontrol efficiency of immobilized *Bacillus subtilis* [33]. Notably, *F. avenaceum* is characterized by relatively high adaptability and partial resistance to antagonistic microorganisms [34], whereas *A. solani* is generally more sensitive, making this pathogen pair suitable for evaluating both moderate (fungistatic) and strong (fungicidal) responses [33].

Antifungal activity was assessed using a spot-on-lawn assay, in which the entire agar surface was uniformly inoculated with fungal spores, followed by placement of polymer films with or without entrapped bacteria. This method ensures immediate contact between the biocontrol agent and the fungal pathogen, allowing direct observation of antagonistic effects such as metabolite diffusion, nutrient competition, and rapid niche colonization. In contrast to dual-culture assays, which rely on indirect interaction, this method emphasizes contact-driven inhibition. Results were recorded after 48 h of incubation, representing early-stage antagonistic interactions.

**Figure 6:**
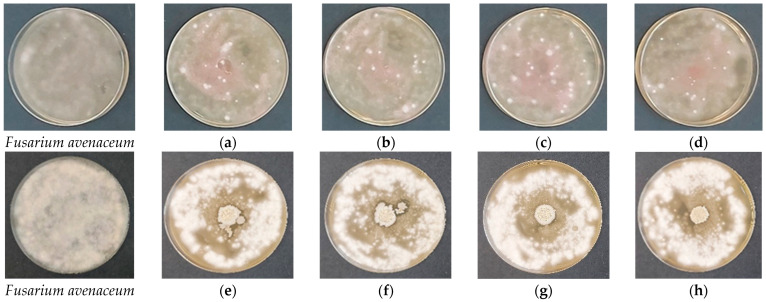
Antifungal activity of freshly prepared cellulose-based films against *Fusarium avenaceum* evaluated by the spot-on-lawn assay after 48 h of incubation at 28 °C. (**a**–**d**) Control films without *Bacillus subtilis*: (**a**) HEC-L; (**b**) HEC-M; (**c**) HEC-H; (**d**) CMC-Na. (**e**–**h**) Films containing immobilized *B. subtilis*: (**e**) HEC-L/*B. subtilis*; (**f**) HEC-M/*B. subtilis*; (**g**) HEC-H/*B. subtilis*; (**h**) CMC-Na/*B. subtilis*.

**Figure 7:**
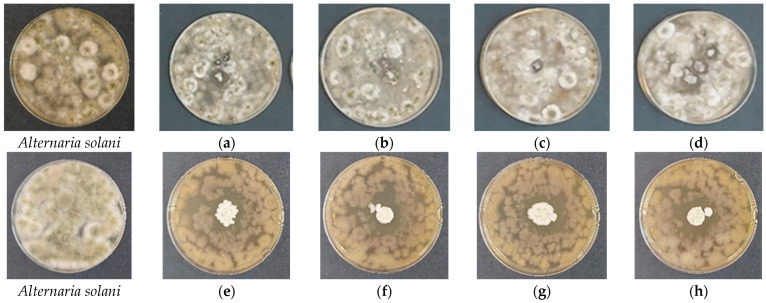
Antifungal activity of freshly prepared cellulose-based films against *Alternaria solani* evaluated by the spot-on-lawn assay after 48 h of incubation at 28 °C. (**a**–**d**) Control films without *Bacillus subtilis*: (**a**) HEC-L; (**b**) HEC-M; (**c**) HEC-H; (**d**) CMC-Na. (**e**–**h**) Films containing immobilized *B. subtilis*: (**e**) HEC-L/*B. subtilis*; (**f**) HEC-M/*B. subtilis*; (**g**) HEC-H/*B. subtilis*; (**h**) CMC-Na/*B. subtilis*.

Control films without *B. subtilis* (Figure 6a–d and Figure 7a–d) showed no intrinsic antifungal activity, as fungal mycelium fully overgrew the film area. This confirms that the cellulose matrices themselves do not possess intrinsic antifungal properties under the tested conditions. Upon hydration, the films swelled rapidly and partially dissolved, consistent with the previously described swelling behavior, particularly for lower-molecular-weight HEC and CMC-Na systems. In contrast, films containing entrapped *B. subtilis* (Figure 6e–h and Figure 7e–h) induced clear growth inhibition zones around the film discs, confirming effective antifungal activity mediated by the bioinoculant. The extent and morphology of inhibition were strongly dependent on both the fungal species and the polymer matrix.

Against *F. avenaceum* (Figure 6e–h), inhibition zones were more pronounced but less uniform, indicating a predominantly fungistatic effect characterized by reduced mycelial density and delayed radial growth rather than complete suppression. The highest inhibition was observed for HEC-H/*B. subtilis*, followed by CMC-Na and HEC-M formulations, while HEC-L exhibited the weakest and least uniform effect. These observations are consistent with SEM and swelling results, where high-molecular-weight HEC forms more compact networks with slower, more controlled release behavior, supporting sustained antifungal activity. Despite inhibition, *F. avenaceum* was still able to develop at the periphery of the Petri dishes, reflecting its relatively high adaptive capacity and partial tolerance to biocontrol stress.

In contrast, *A. solani* (Figure 7e–h) exhibited uniform and well-defined inhibition zones across all formulations, indicating a predominantly fungicidal effect with complete suppression of fungal growth within the inhibition area. Unlike *F. avenaceum*, no clear dependence on polymer molecular weight was observed, suggesting that *A. solani* is highly sensitive to antimicrobial metabolites produced by *B. subtilis*, regardless of the delivery matrix.

An additional observation was the formation of dry, smooth biofilm-like bacterial colonies on the film surface, typical of *Bacillus subtilis*. This confirms active bacterial metabolism and biofilm development, a key trait of plant growth-promoting rhizobacteria (PGPR). The polymer matrix influences this behavior: denser HEC-H matrix likely provides a more stable microenvironment, supporting sustained bacterial activity and controlled metabolite diffusion, whereas less cohesive systems (HEC-L and HEC-M) promote faster but less controlled release. The observed antifungal performance is strongly correlated with the structural and swelling properties of the films. As shown by SEM analysis, high-molecular-weight HEC produces compact and homogeneous networks, enhancing spore immobilization and controlled release. Swelling studies further demonstrated that HEC-H maintains gel-like integrity for extended periods, enabling sustained hydration and gradual activation of bacterial spores. In contrast, lower-molecular-weight systems undergo rapid swelling and disintegration, resulting in faster but less spatially controlled release of bioactive compounds.

**Figure 8:**
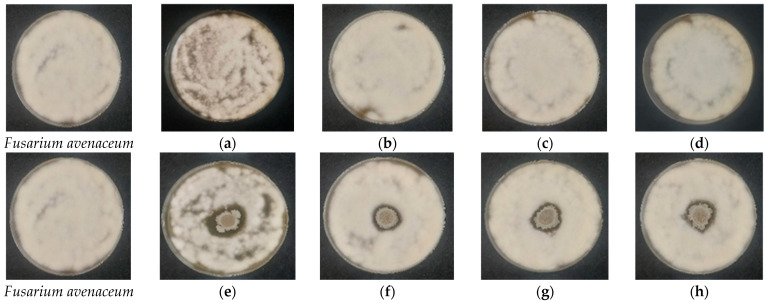
Antifungal activity of 12-month stored cellulose-based films against *Fusarium avenaceum* evaluated by the spot-on-lawn assay following 48 h of incubation at 28 °C. (**a**–**d**) Control films without *Bacillus subtilis*: (**a**) HEC-L; (**b**) HEC-M; (**c**) HEC-H; (**d**) CMC-Na. (**e**–**h**) Films containing immobilized *B. subtilis*: (**e**) HEC-L/*B. subtilis*; (**f**) HEC-M/*B. subtilis*; (**g**) HEC-H/*B. subtilis*; (**h**) CMC-Na/*B. subtilis*.

**Figure 9:**
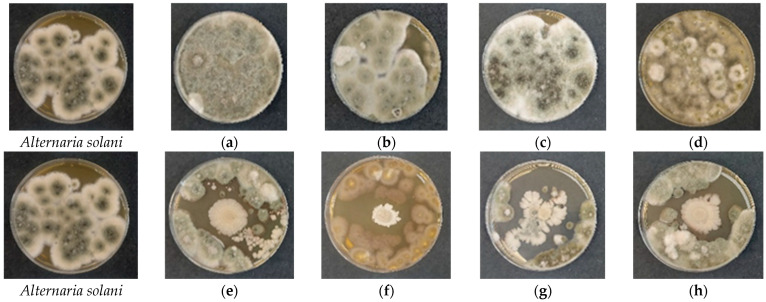
Antifungal activity of 12-month stored cellulose-based films against *Alternaria solani* evaluated by the spot-on-lawn assay following 48 h of incubation at 28 °C. (**a**–**d**) Control films without *Bacillus subtilis*: (**a**) HEC-L; (**b**) HEC-M; (**c**) HEC-H; (**d**) CMC-Na. (**e**–**h**) Films containing immobilized *B. subtilis*: (**e**) HEC-L/*B. subtilis*; (**f**) HEC-M/*B. subtilis*; (**g**) HEC-H/*B. subtilis*; (**h**) CMC-Na/*B. subtilis*.

The antifungal activity of the biohybrid films after 12 months of storage is presented in Figure 8 and Figure 9. The results confirm that the entrapped *Bacillus subtilis* remains viable and biologically active, as evidenced by the formation of distinct inhibition zones against both tested pathogens.

Against *Fusarium avenaceum* (Figure 8), inhibition zones were still clearly visible for all formulations. However, they were narrower and more sharply defined compared to freshly prepared films. The inhibition pattern shifted from diffuse, low-density (fungistatic) zones to more localized regions with reduced or absent fungal growth. This behavior is consistent with a more spatially restricted release of bioactive compounds after storage. Despite the reduction in zone diameter, the persistence of inhibition confirms retained antifungal functionality.

In the case of *Alternaria solani* (Figure 9), all films continued to exhibit strong antifungal activity, characterized by well-defined inhibition zones with complete suppression of fungal growth. The overall morphology of the inhibition zones remained comparable to that of freshly prepared samples, indicating preservation of fungicidal activity over time. Minor differences in zone uniformity and occasional spore dispersion around the film were observed, likely related to increased film fragility after storage. Importantly, the bacterial colonies emerging from the films retained their typical morphology, indicating that *B. subtilis* remained viable, capable of germination, and progressed toward sporulation after incubation. This confirms that neither entrapment nor long-term storage induced detectable physiological stress affecting bacterial development.

These results demonstrate that cellulose-based matrices effectively preserve the viability and functional activity of *B. subtilis* over prolonged storage periods. The observed changes in inhibition patterns are more likely associated with alterations in polymer physical properties, such as reduced swelling capacity and increased brittleness, rather than any loss of microbial functionality.

These qualitative observations are confirmed by quantitative analysis (Table 1). For *A. solani*, all formulations showed similar inhibition zones (29.1–32.5 mm initially), with no statistically significant differences (*p* = 0.18), confirming uniform fungicidal activity. In contrast, *F. avenaceum* showed strong dependence on polymer composition, with inhibition zones ranging from 33.1 ± 2.1 mm (HEC-L) to 44.8 ± 2.4 mm (HEC-H). One-way ANOVA revealed highly significant differences (*p* < 0.001), and post hoc analysis confirmed that HEC-H exhibited significantly higher activity than all other formulations (*p* < 0.01), while no significant difference was observed between HEC-L and HEC-M.

After 12 months of storage, antifungal activity was retained in all systems, although with reduced magnitude. For *A. solani*, inhibition zones remained comparable (27.4–34.4 mm), with no significant differences (*p* > 0.05), confirming long-term stability of fungicidal activity. For *F. avenaceum*, inhibition zones decreased (19.1–24.4 mm), and statistically significant differences between formulations were observed (*p* < 0.05). Notably, HEC-M exhibited significantly lower activity compared to HEC-L and CMC-Na (*p* < 0.01), while HEC-H maintained intermediate performance.

A shift in inhibition pattern was also observed after storage, where broader fungistatic zones transitioned into narrower but more sharply defined inhibition areas. This suggests a change in diffusion behavior of active compounds, likely associated with structural aging of the polymer matrix, including increased brittleness, and reduced swelling capacity. As a result, diffusion becomes more localized, leading to higher effective concentrations near the film–agar interface.

The observed matrix-dependent behavior may also have practical relevance under agricultural conditions. Rapidly hydrating systems such as CMC-Na could be advantageous in applications requiring fast release of viable *Bacillus subtilis* cells and antimicrobial metabolites, for example on moist leaf surfaces or immediately after irrigation, where rapid colonization of infection sites is desirable. In contrast, the greater structural persistence and sustained release behavior of HEC-H may be more suitable for soil or rhizosphere environments, where prolonged microbial survival and gradual metabolite diffusion are beneficial under fluctuating moisture conditions. Such differences suggest that polymer selection can be tailored according to the intended route of application, environmental humidity, and required duration of biocontrol activity.

These results demonstrate that polymer composition and molecular weight critically influence antifungal performance, particularly against *F. avenaceum*, where matrix-dependent differences are pronounced. In contrast, *A. solani* exhibits similar susceptibility across all formulations. Importantly, long-term storage does not compromise biological activity, confirming that entrapped *B. subtilis* remain viable and functional, while observed changes in inhibition patterns reflect time-dependent modifications in polymer structure and release kinetics.

## 3. Conclusions

This study demonstrates that cellulose derivative films can be effectively engineered as functional biohybrid carriers for the immobilization and delivery of *Bacillus subtilis* for antifungal applications. The molecular weight and chemical nature of the polymer matrix were identified as key determinants of film morphology, swelling behavior, mechanical properties, and biological performance.

High-molecular-weight HEC formed dense and mechanically robust networks that improved structural integrity, controlled swelling, and sustained microbial activity. In contrast, low- and medium-molecular-weight HEC provided more rapidly swelling and mechanically flexible systems, while CMC-Na exhibited high stiffness but limited structural stability due to its polyelectrolyte character. These differences modulated the microenvironment surrounding the entrapped bacteria and influenced the diffusion of bioactive compounds. In this context, the cellulose matrix functions primarily as a biocompatible scaffold that maintains spore viability and regulates their activation and release.

All biohybrid films preserved biological functionality after 12 months of storage, confirming the long-term stability and viability of immobilized spores under ambient conditions. Control films without *B. subtilis* showed no antifungal activity, confirming that the cellulose-derived matrices do not exhibit intrinsic antifungal properties. Antifungal assays demonstrated effective inhibition of both *Fusarium avenaceum* and *Alternaria solani*, with pathogen-dependent responses reflecting differences in susceptibility and matrix-mediated release behavior. Accordingly, the antifungal effect is attributable to *B. subtilis* and its metabolites, while the polymer matrix acts as a protective and release-controlling carrier. Importantly, high-molecular-weight HEC provided the most balanced performance, combining mechanical stability with sustained antifungal activity. These findings highlight the importance of polymer design in tailoring structure–property–function relationships in biohybrid materials and support the potential of cellulose-based systems as biodegradable and tunable platforms for microbial delivery in sustainable plant protection.

## 4. Materials and Methods

### 4.1. Materials

Film-forming solutions were prepared using cellulose derivatives, including 2-hydroxyethyl cellulose (HEC) with low (HEC-L, average M_v_ ~90,000 g/mol), medium (HEC-M, average M_w_ ~250,000 g/mol), and high (HEC-H, typical M_v_ ~1,300,000 g/mol) molecular weights. Sodium carboxymethyl cellulose (CMC-Na, average M_w_ ~250,000 g/mol, degree of substitution ~0.9) was used as a reference polymer. All polysaccharides were supplied by Sigma-Aldrich (Darmstadt, Germany) and used without further purification. Analytical-grade reagents CH_3_COOH, NaOH, KH_2_PO_4_, Na_2_HPO_4_, NaHCO_3_, and Na_2_CO_3_ for buffer preparation were obtained from Merck (Darmstadt, Germany). Buffer solutions with pH values of 4.0, 7.0, and 9.0 were prepared using appropriate buffering systems and used immediately after preparation.

The microbial strains were obtained from the private collection of Biodynamica Ltd. (Plovdiv, Bulgaria). The biocontrol agent is identified as *Bacillus subtilis* strain A9.2AG. The phytopathogenic fungi *Alternaria solani* and *Fusarium avenaceum* were also sourced from the same collection.

The bacterial strain *Bacillus subtilis* (Biodinamika Ltd., Plovdiv, Bulgaria) was cultivated in Tryptic Soy Broth (TSB) at 28 °C under continuous agitation (197 rpm) until sporulation was achieved. The spores were harvested by centrifugation (6000 rpm, 4 °C, and 15 min) and washed twice with sterile distilled water after incubation for 72 h (or 5 days, depending on the experiment). The resulting suspension was adjusted to a final concentration of 1 × 10^10^ spores/mL.

The phytopathogenic fungi *Fusarium avenaceum* and *Alternaria solani* were maintained on Potato Dextrose Agar (PDA) under standard laboratory conditions. For experimental use, 5-mm-diameter agar plugs containing actively growing mycelium were transferred onto fresh PDA plates and incubated at 28 °C until full colony development was achieved. Under these conditions, both phytopathogens exhibited robust vegetative growth, accompanied by abundant sporulation and the formation of viable conidia.

### 4.2. Solution Casting of Cellulose-Based Films

Films based on cellulose derivatives were prepared by solution casting from aqueous solutions of 2-hydroxyethyl cellulose (HEC) and sodium carboxymethyl cellulose (CMC-Na). HEC solutions of different molecular weights (HEC-L, HEC-M, and HEC-H) were prepared at a concentration of 1% (*w*/*v*) by dissolving the polymers in purified water under continuous magnetic stirring at ambient temperature until homogeneous viscous solutions were obtained. CMC-Na solutions were prepared following the same procedure. For film formation, equal volumes of the prepared polymer solutions were poured into Petri dishes and allowed to dry at room temperature under ambient conditions until constant weight was reached. All films were cast on a leveled surface to ensure uniform thickness and controlled solvent evaporation.

For bacterial immobilization, an appropriate volume of *Bacillus subtilis* suspension was added into each polymer solution prior to casting, followed by gentle mixing to ensure uniform distribution. A ratio of 5 mL of spore suspension per 1 g of polymer was applied to achieve the desired microbial loading. The resulting mixtures were then cast into Petri dishes and dried under the same conditions.

After drying, the films were carefully peeled off from the casting surface and stored under ambient conditions until further characterization.

### 4.3. Films Characterization

Surface morphology of the films was examined using a scanning electron microscope (SEM, JSM-5510, JEOL Ltd., Tokyo, Japan). Prior to imaging, samples were sputter-coated with a thin gold layer using a JEOL JFC-1200 fine coater (JEOL Co. Ltd., Tokyo, Japan) to improve electrical conductivity.

The dynamic viscosity of the polymer solutions was measured using a Brookfield DV-II+ Pro viscometer (Brookfield, Middleboro, MA, USA) equipped with a cone–plate geometry (CPE-52 spindle). Measurements were performed at 25 ± 0.1 °C using a thermostated sample cup to ensure temperature stability.

Mechanical properties of the films were evaluated using an Instron 3344 universal testing machine (Instron, Norwood, MA, USA) equipped with a 50 N load cell. Film samples were cut into rectangular strips (20 mm × 60 mm), and thickness was measured using a digital thickness gauge (FD 50, Käfer GmbH, Villingen-Schwenningen, Germany). Tensile tests were conducted at a constant crosshead speed of 10 mm/min at room temperature. Stress–strain curves were recorded using Bluehill Universal software (v. 3.11), and Young’s modulus, tensile strength, and elongation at break were determined. At least 10 specimens per sample were analyzed, and mean values are reported.

The swelling behavior of the films based on cellulose derivatives (HEC-L, HEC-M, HEC-H, and CMC-Na) in acidic, neutral, and alkaline media was evaluated using a gravimetric method in buffer solutions with pH values of 4.0, 7.0, and 9.0 (ionic strength I = 0.1) at 25 °C. Dry film specimens with known initial mass were placed in the corresponding buffer solutions, incubated and maintained under these conditions until swelling equilibrium was achieved. At selected time intervals, the samples were withdrawn, carefully blotted to remove residual surface moisture, and weighed. Measurements continued until a constant mass was obtained. The swelling degree (S, %) was calculated according to Equation (1):

(1) $$S,\;\%\;=\;\frac{w_{s}-w_{d}}{w_{d}}\times 100,$$

where w_s_ is the weight of the swollen film and w_d_ is the initial dry weight. All measurements were performed in triplicate, and average values are reported.

### 4.4. Microbiological Purity, Viability, and Long-Term Stability

Microbiological purity of the prepared films was initially verified using control samples without incorporated bacterial spores, ensuring the absence of contamination during the preparation process.

The viability of immobilized *Bacillus subtilis* was evaluated based on their capacity to germinate and proliferate on a solid nutrient medium. Film discs (10 mm in diameter) were aseptically placed onto Tryptic Soy Agar (TSA) plates and incubated at 28 °C. The development of bacterial colonies was monitored at 18, 48, and 72 h to assess spore germination and subsequent growth.

To examine long-term stability, the films were stored at room temperature (23 ± 2 °C; 45 ± 5% relative humidity) for a period of 12 months. After storage, viability was assessed by placing film discs onto fresh TSA plates and incubating them at 28 °C for 48 h. The presence and intensity of bacterial growth were used as indicators of retained viability and metabolic activity after prolonged storage.

### 4.5. Antifungal Activity of Biohybrid Cellulose-Derived Films

The antifungal performance of the cellulose-derived films was evaluated using a dual-culture (spot-on-lawn) assay against the phytopathogens *Fusarium avenaceum* and *Alternaria solani*, following methodologies described in previous studies [35]. A fungal spore suspension, adjusted to a concentration of approximately 1 × 10^2^ spores/mL, was evenly distributed over the surface of Potato Dextrose Agar (PDA) plates and allowed to dry under aseptic conditions. Film discs (10 mm in diameter), either containing or lacking *Bacillus subtilis*, were then placed at the center of the inoculated plates. The samples were incubated at 28 °C for 48 h. Antifungal activity was assessed by measuring the diameter of the clear or inhibition zones formed around each film disc, representing the region of suppressed fungal growth. In addition to inhibition zone measurements, the morphology of the fungal mycelium at the film–agar interface was closely examined to identify any structural alterations or growth retardation induced by the biohybrid matrix.

### 4.6. Statistical Analysis

All experiments were performed in quintuplicate (*n* = 5), and the results are presented as mean values ± standard deviation (SD). To ensure high precision in evaluating antifungal activity, two perpendicular diameters (d_1_ and d_2_) of the inhibition zones were measured at right angles for each replicate. The average value used for subsequent analysis was calculated as follows:

(2) $$X_{\mathrm{replicate}}\;=\;\frac{d_{1}+d_{2}}{2},$$

Given the small sample size, variability was calculated using the sample standard deviation (s), applying Bessel’s correction (n − 1) to provide a less biased estimate of the population variance:

(3) $$s=\sqrt{\frac{{\Sigma (x_{i}-\bar{x})}^{2}}{n-1}},$$

where x_i_ represents the individual measurements of the inhibition zones (categorized as fungicidal for *A. solani* and fungistatic for *F. avenaceum*), $\bar{x}$ denotes the corresponding arithmetic mean, and (n − 1) represents the degrees of freedom.

Statistical significance among the various cellulose derivative formulations was assessed using one-way analysis of variance (ANOVA). To identify specific pairwise differences and compare the efficacy of the biohybrid films, Tukey’s Honestly Significant Difference (HSD) post hoc test was applied. A *p*-value < 0.05 was considered statistically significant. All statistical analyses and data visualization were performed using GraphPad Prism (version 5.0, GraphPad Software Inc., San Diego, CA, USA).

## Figures and Tables

**Table 1:** Antifungal activity of biohybrid films containing *B. subtilis*, expressed as inhibition zone diameters (mm ± SD, *n* = 5), evaluated immediately after preparation and after 12 months of storage.

Film Type	*F. avenaceum* Zone (mm)	*F. avenaceum* Zone (mm) After 12 Months	*A. solani* Zone (mm)	*A. solani* Zone (mm) After 12 Months
HEC-L/*B. subtilis*	33.1 ± 2.1 ^b^	24.4 ± 4.3 ^b^	32.5 ± 1.1 ^a^	32.0 ± 3.2 ^a^
HEC-M/*B. subtilis*	36.1 ± 4.4 ^b^	19.1 ± 1.9 ^c^	30.5 ± 2.4 ^a^	27.4 ± 3.8 ^a^
HEC-H/*B. subtilis*	44.8 ± 2.4 ^a^	21.5 ± 1.4 ^b,c^	29.1 ± 3.5 ^a^	33.6 ± 4.3 ^a^
CMC-Na/*B. subtilis*	36.9 ± 4.3 ^b^	23.2 ± 2.1 ^b^	32.2 ± 1.8 ^a^	34.4 ± 3.4 ^a^

Different superscript letters (a–c) within the same column indicate statistically significant differences (*p* < 0.05; one-way ANOVA followed by Tukey’s test).

## Data Availability

The data are contained within this article.

## Supplementary Materials

The following supporting information can be downloaded at: https://www.mdpi.com/article/10.3390/gels12050366/s1, Figure S1: Photographs of cellulose derivative films. (**a**–**d**) Control films without *Bacillus subtilis*: (**a**) HEC-L; (**b**) HEC-M; (**c**) HEC-H; (**d**) CMC-Na. (**e**–**h**) Films containing immobilized *Bacillus subtilis*: (**e**) HEC-L/*B. subtilis*; (**f**) HEC-M/*B. subtilis*; (**g**) HEC-H/*B. subtilis*; (**h**) CMC-Na/*B. subtilis*; Figure S2: SEM micrographs of cellulose derivative films: (**a**) HEC-L; (**b**) HEC-M; (**c**) HEC-H; (**d**) CMC-Na.

## Abbreviations

The following abbreviations are used in this manuscript:

HEC-L	2-Hydroxyethyl cellulose with low molecular weight
HEC-M	2-Hydroxyethyl cellulose with medium molecular weight
HEC-H	2-Hydroxyethyl cellulose with high molecular weight
CMC-Na	Sodium carboxymethyl cellulose
