# Supplementary material for: Tailoring Cellulose Derivative Gel Matrices for Bacillus subtilis Delivery: Effects of Polymer Molecular Weight on Stability and Biocontrol
Source: Gels. 2026 Apr 27;12(5):366. doi: 10.3390/gels12050366 (PMC13206064; doi:10.3390/gels12050366)
Supplement: Supplementary file 1 [file gels-12-00366-s001.zip › gels-4266874-supplementary.pdf]

gels

Supplementary Materials

Tailoring Cellulose-Derivative Gel Matrices for *Bacillus subtilis* Delivery: Effects of Polymer Molecular Weight on Stability and Biocontrol

Petya Tsekova [1,2], Nasko Nachev [1], Iliyana Valcheva [3], Donka Draganova [3], Mariya Spasova [1] and Olya Stoilova [1,2,*]

1 Laboratory of Bioactive Polymers, Institute of Polymers, Bulgarian Academy of Sciences, 1113 Sofia, Bulgaria; cekovapetya@polymer.bas.bg (P. T.); nachev_n@polymer.bas.bg (N.N.); mspasova@polymer.bas.bg (M.S.); stoilova@polymer.bas.bg (O.S.)
2 National Centre of Excellence Mechatronics and Clean Technologies, 8 Blvd. Kliment Ohridski, 1000 Sofia, Bulgaria; cekovapetya@polymer.bas.bg (P.T.); stoilova@polymer.bas.bg (O.S.)
3 Biodinamika Ltd., 4000 Plovdiv, Bulgaria; valchevailiana1@gmail.com (I.V.); donkadraganova@gmail.com (D.D.)
* Correspondence: stoilova@polymer.bas.bg

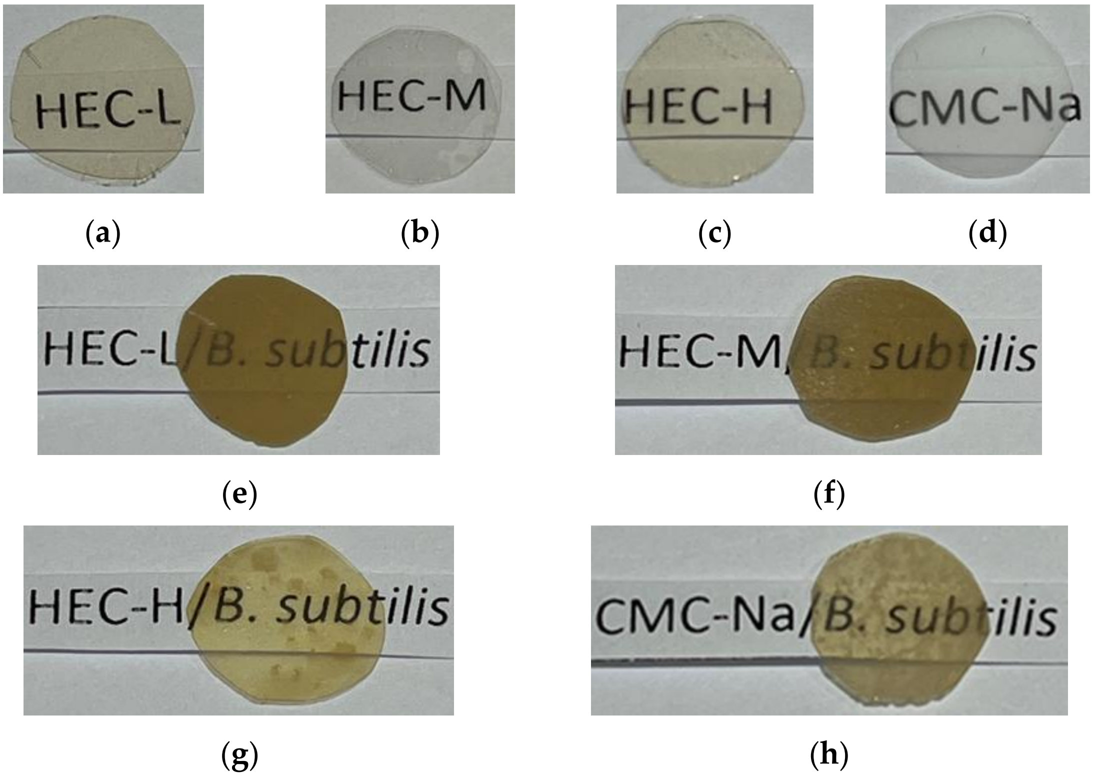

Figure S1. Photographs of cellulose-derivative films. (**a–d**) Control films without *Bacillus subtilis*: (**a**) HEC-L; (**b**) HEC-M; (**c**) HEC-H; (**d**) CMC-Na. (**e–h**) Films containing immobilized *Bacillus subtilis*: (**e**) HEC-L/*B. subtilis*; (**f**) HEC-M/*B. subtilis*; (**g**) HEC-H/*B. subtilis*; (**h**) CMC-Na/*B. subtilis*.

 https://doi.org/10.3390/gels12050366

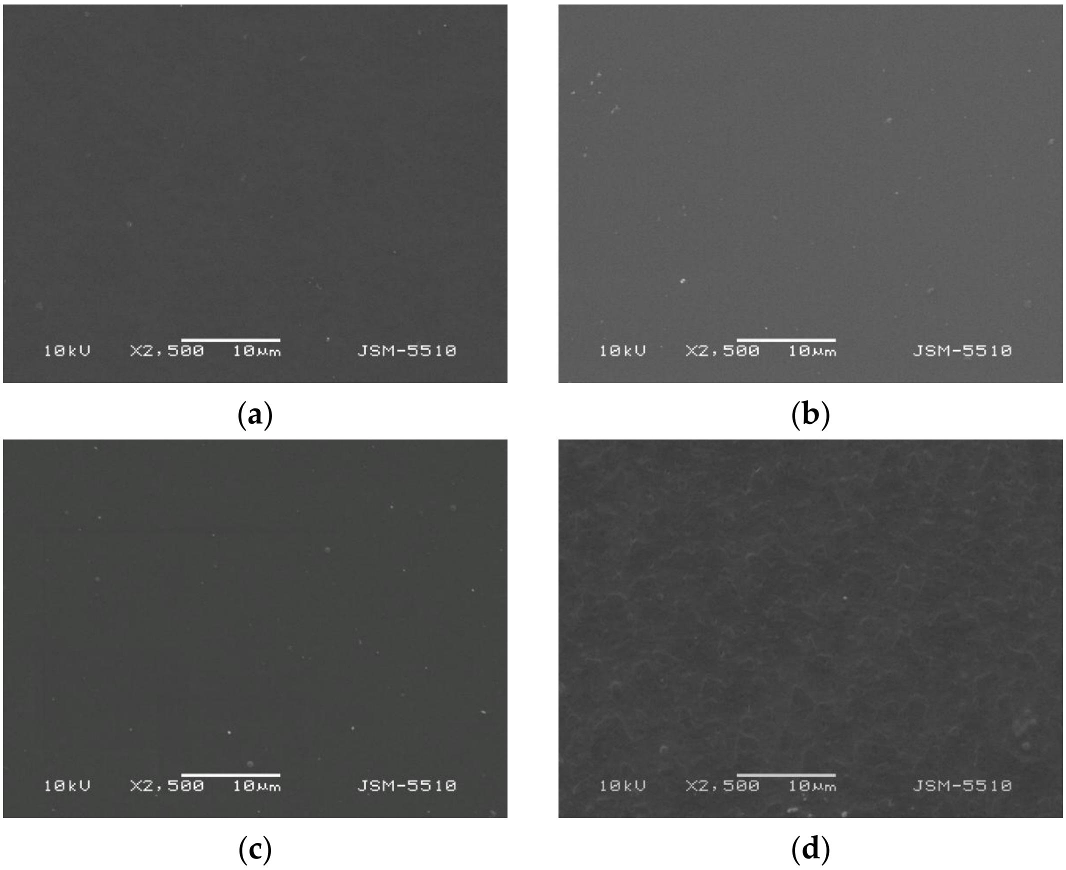

Figure S2. SEM micrographs of cellulose-derivative films: (**a**) HEC-L; (**b**) HEC-M; (**c**) HEC-H; (**d**) CMC-Na.
